# Effects of Aspirin and Prednisone on Platelet Function and Thromboxane Synthesis in Healthy Dogs

**DOI:** 10.3389/fvets.2019.00393

**Published:** 2019-11-15

**Authors:** John M. Thomason, Allison P. Mooney, Joshua M. Price, Jacqueline C. Whittemore

**Affiliations:** ^1^The Department of Clinical Sciences, College of Veterinary Medicine, Mississippi State University, Mississippi State, MS, United States; ^2^The Department of Small Animal Clinical Sciences, College of Veterinary Medicine, University of Tennessee, Knoxville, Knoxville, TN, United States; ^3^The Office of Information Technology, College of Veterinary Medicine, University of Tennessee, Knoxville, Knoxville, TN, United States

**Keywords:** corticosteroid, glucocorticoid, antiplatelet, thromboprophylaxis, immune-mediated hemolytic anemia, acetylsalicylic acid

## Abstract

Glucocorticoid administration is a risk factor for thromboembolism in hypercoagulable dogs, and it is unknown if aspirin counteracts glucocorticoid-induced hypercoagulability. The objective was to determine the effects of sustained aspirin and prednisone administration on platelet function and thromboxane synthesis. Our hypothesis was that aspirin would consistently inhibit platelet function and thromboxane synthesis when administered with or without prednisone. In 24 healthy dogs, platelet aggregometry and urine 11-dehydro-thromboxane-B_2_ (11-dTXB_2_)-to-creatinine ratios were measured on days 0, 14, and 28. Dogs were administered placebos, aspirin (2 mg/kg/d), prednisone (2 mg/kg/d), or prednisone/aspirin combination therapy PO for 28 days in a randomized double-blinded study. Aspirin response was based on a >25% reduction in platelet aggregation compared to pre-treatment values. Results were compared using mixed model, split-plot repeated measures ANOVAs. *P* < 0.05 was considered significant. AUC differed significantly by time [*F*_(2,40)_ = 10.2, *P* < 0.001] but not treatment or treatment-by-time. On day 14, 2 dogs were aspirin responders (aspirin, 1; placebo, 1). On day 28, 3 dogs were aspirin responders (aspirin, 2; prednisone/aspirin, 1). Urine 11-dTXB_2_-to-creatinine ratios differed significantly by group [*F*_(3,20)_ = 3.9, *P* = 0.024] and time [*F*_(2,40)_ = 8.7, *P* < 0.001), but not treatment-by-time. *Post-hoc* analysis revealed significant differences between aspirin and placebo groups (*P*=0.008), aspirin and prednisone/aspirin groups (*P* = 0.030), and placebo and prednisone groups (*P* = 0.030). In healthy dogs, sustained aspirin, prednisone, and combination therapy do not inhibit platelet aggregation, and when used as individual therapies, aspirin and prednisone decreased thromboxane synthesis. Additional studies using varied platelet function methodologies in hypercoagulable dogs are necessary.

## Introduction

Glucocorticoids are the mainstay of therapy for immune-mediated disorders including immune-mediated hemolytic anemia (IMHA), immune-mediated thrombocytopenia, inflammatory bowel disease, and immune-mediated polyarthritis. Dogs diagnosed with IMHA have a historically high mortality rate (50–70%) (1, 2) with one of the most common causes of death being thromboembolism (1–3). The true incidence of thromboembolism is unknown but is estimated to be as high as 80% (3). The mechanisms that cause thrombus formation in dogs with IMHA are multifactorial and include endothelial damage, an imbalance between pro- and anticoagulant factors, and increased platelet activation (2, 4, 5).

Standard treatment for dogs with IMHA is multimodal and includes immunosuppression, thromboprophylaxis, and supportive care (1, 6). Glucocorticoids, such as prednisone, are the most commonly used immunosuppressive agent for the treatment of IMHA in dogs. Unfortunately, glucocorticoid administration can result in hypercoagulability in healthy dogs (7–9), and it has been identified as a risk factor for thromboembolism in clinical patients (8, 10, 11). Thromboprophylactic medications commonly are administered to dogs with IMHA to prevent thromboembolic complications (5, 12). Although anticoagulant therapy is commonly recommended as the preferred thromboprophylactic therapy (5), many of these medications are either cost prohibitive and/or administered via injection, leaving oral aspirin as one of the most affordable options for long-term prophylactic therapy. Weinkle et al. (1) reported longer short and long-term survival times for dogs with IMHA treated with ultra-low dose aspirin (0.5 mg/kg/d), even though the dogs treated with ultra-low dose aspirin had more risk factors for increased mortality. Aspirin irreversibly inhibits platelet function by decreasing thromboxane A_2_ (TXA_2_) synthesis (13). Primarily produced by activated platelets, TXA_2_ triggers vasoconstriction, causes platelet activation, and enhances platelet aggregation (14).

Although an anti-inflammatory aspirin dose (10 mg/kg q12h) reliably inhibits TXA_2_ synthesis and platelet function (15), it causes gastric ulceration and renal side effects in dogs (16, 17). Only one-third of healthy dogs administered low-dose aspirin (1 mg/kg/d) have consistent inhibition of platelet function (14, 18, 19), whereas a slightly higher aspirin dose (2 mg/kg/d) consistently inhibited platelet function in one short-term study of healthy dogs (14). However, it is unknown if low-dose aspirin counteracts glucocorticoid-induced hypercoagulability or, alternatively, if glucocorticoid-induced hypercoagulability offsets the antiplatelet effects of aspirin. Previous studies assessing the combined hemodynamic effects of immunosuppressive doses of prednisone and aspirin in healthy dogs used thromboelastography to assess hypo- or hypercoagulable profile, but platelet function was not specifically evaluated (7, 8). Additionally, these studies used an aspirin dose of 0.5 mg/kg/d, which since has been found to be inadequate for consistent platelet inhibition in healthy dogs (14). Finally, these studies were limited in duration to 6 and 14 days of therapy.

The objective of this randomized-controlled double-blinded study was to determine the platelet function and thromboxane synthesis in healthy dogs receiving placebo, aspirin (2 mg/kg/d), prednisone (2 mg/kg/d), or combination prednisone and aspirin therapy. Our hypothesis was that sustained administration of aspirin would consistently inhibit platelet function and thromboxane synthesis when administered singly or with concurrent immunosuppressive doses of prednisone.

## Materials and Methods

### Study Population

Blood samples for this study were collected from 24 healthy dogs from the University of Tennessee, College of Veterinary Medicine teaching and research colony during a related study assessing gastrointestinal effects of sustained aspirin and prednisone therapy (20). Dogs were determined to be healthy based on lack of clinical signs of disease and lack of abnormalities on physical examination and CBC, serum chemistry panel, urinalysis, urine protein:creatinine ratio, and fecal flotation with direct smear (20). Animal use was approved by the University of Tennessee, College of Veterinary Medicine Institutional Animal Care and Use Committee (protocol number 2283) and was in compliance with the requirements of a facility accredited by the American Association for Accreditation of Laboratory Animal Care.

A sample size calculation was performed using data from a previous study that measured inhibition of platelet function in healthy dogs receiving aspirin at a 2 mg/kg/d dosage (14). Assuming a one-way ANOVA to detect main effect treatment differences and a standard deviation of 15.6%, 6 dogs per treatment group would be needed to have 85% power to find a difference of 25% in platelet aggregation significant with an alpha of 0.05. A reduction of 25% in canine platelet aggregation while receiving aspirin indicates a response to therapy (19). The power analysis was performed using SAS 9.4 release TS1M5, SAS Institute Inc., Cary NC.

### Study Design

The dogs were stratified by age and assigned to 1 of 4 treatment groups using a random number sequence generator (https://www.random.org, accessed May 16, 2017). The treatment groups include: (1) placebo, (2) aspirin (2 mg/kg, PO, q24h) plus placebo, (3) prednisone (2 mg/kg, PO, q24h) plus placebo, and (4) aspirin (2 mg/kg, PO, q24h) and prednisone (2 mg/kg, PO, q24h). Dogs in the placebo group received 2 placebo capsules once daily, while dogs in groups 2 and 3 were administered 1 placebo capsule. Using compounding standards provided by the United States Pharmacopeia (USP), commercially-available aspirin tablets (Rugby Laboratories, Livonia MI) were compounded into capsules by the College's pharmacy. Prednisone was administered using commercially-available prednisone tablets (West-Ward Pharmaceuticals Corp., Eatentown NJ). The placebo capsules (LetCo Medical, Decatur AL) were assembled by the College's pharmacy using lactose. All treatments were administered in small meatballs once daily prior to feeding by an individual blinded to the individual treatments and groups.

The study involved three periods: acclimation, baseline, and treatment. The acclimation period occurred on days−13 to−7, during which dogs were administered fenbendazole (50 mg/kg/d, PO, days−13 to−9) and ivermectin (200 μg/kg SQ once, day−13). As part of routine colony prophylaxis, dogs also received imidacloprid and moxidectin (Advantage Multi® for dogs, Bayer HealthCare, LLC, Shawnee Mission KS), according to manufacturer's instructions. The baseline period occurred from days −6 to 0, and the treatment period was from days 1 to 28.

Blood and urine samples were collected at the conclusion of baseline and on days 14 and 28 and within 3 h of drug administration. Blood was collected by jugular venipuncture with a 20-gauge needle directly into Vacutainer tubes containing EDTA (hematocrit and manual platelet count) and hirudin (aggregometry). For platelet aggregometry, the tube of blood was held at room temperature without disturbance until analysis, and all samples were analyzed within 4 h of collection. Approximately 2–5 mL of urine was collected via free catch, catheterization, or cystocentesis using a 22-gauge 1.5-inch needle with a 6 mL syringe; urine was stored at −80°C for 6–7 months for later analysis.

### Hematologic Testing

Complete blood counts with manual platelet counts were performed at each time point by a commercial diagnostic laboratory (Methodology Automated Blood Analyzer, Antech Diagnostics, Fountain Valley CA).

Impedance platelet aggregometry was performed with a multiple electrode aggregometer (Multiplate® Analyzer, Verum Diagnostica GmbH, Munich, Germany) that has been previously validated for use in dogs (21–23). Per the manufacturer's instructions (Multiplate® Analyzer Manual, Verum Diagnostica GmbH, Munich, Germany), each blood sample was inverted 3–5 times and then transferred into a single-use test cell containing a dual sensor unit, a Teflon-coated magnetic stir bar, and warmed saline. Within the dual sensor unit, the electrical resistance between the sensor wires was recorded. Aggregation was assessed using arachidonic acid at a final concentration of 0.5 mM (ASPI test, Roche Diagnostics GmbH, Mannheim, Germany) (24) at a temperature of 37°C for 6 min. Platelet aggregation was analyzed and recorded as area under the curve (AUC). Each dual sensor unit generated 2 separate results, which were averaged to yield a single AUC value at each time point. For the 2 measurements that were used to create the final AUC value, a deviation from the mean was calculated. All samples were analyzed within 4 h of blood collection. A dog was considered to be an aspirin-responder if there was a >25% reduction in AUC compared to pre-treatment values (19).

### Urine 11-dehydro-thromboxane B_2_ (11-dTXB_2_) Ratios

The urine concentration of 11-dTXB_2_ (a stable TXA_2_ metabolite) was measured using an ELISA kit (11-dTXB_2_ EIA kit-Monoclonal, Cayman Chemical Co, Ann Arbor MI) that has been previously validated in dogs (25, 26). Samples were analyzed per the manufacturer's instructions. Samples were thawed to room temperature, diluted with assay buffer, and analyzed at a wavelength of 412 nm using a plate reader (SpectraMax M5 Multi-Mode Microplate Reader, Molecular Devices, Sunnyvale CA). Samples were analyzed in triplicate and the results were averaged. The concentrations of 11-dTXB_2_ were reported in picograms per milliliter of urine. Urine creatinine concentration was measured using a biochemistry analyzer (ACE Alera® Clinical Chemistry System, Alfa Wasserman, Inc., West Caldwell NJ), and a urinary 11-dTXB_2_-to-creatinine ratio was calculated.

### Statistical and Data Analysis

Descriptive statistics were generated for relevant clinical and clinicopathologic parameters. Additionally, box-and-whisker plots were analyzed for the presence of outliers. Platelet count, hematocrit, AUC, AUC deviation from the mean, and 11-dTXB_2_:creatinine ratio were compared using split-plot repeated measures ANOVAs that included fixed effects of treatment group, sampling time, and the treatment-by-time interaction (27). The repeated measure of time was accounted for in a repeated statement. Dog within treatment group was included as a random effect. Fisher's Least Significant Difference (LSD) was used to perform *post-hoc* analyses. The Shapiro-Wilk test of normality and QQ plots of the residuals were evaluated for each marker to confirm the assumption of normally distributed residuals had been met. Model assumptions regarding equality of variances were verified with Levene's Test for Equality of Variances. Studentized residual diagnostics were performed to evaluate each mixed model for the presence of outliers. Differences in marginal means were determined for markers with significant main effect or interaction terms. Non-normally distributed data were rank-transformed, as necessary, to meet underlying statistical assumptions. Fisher's exact test was performed to assess the relationship between treatment and AUC response on days 14 and 28. Statistical computer programs (MedCalc 15.8 MedCalc Software, Ostend, Belgium; SAS 9.4 release TS1M5, SAS Institute Inc., Cary NC) were used for all analyses and a *P*-value of < 0.05 was considered significant.

## Results

### Study Population

Details of the study population have been reported elsewhere (20). Briefly, there were 9 intact females, 8 intact males, and 7 neutered males. There were 15 beagles and 9 mixed breed hounds, which were evenly distributed among the treatment groups. Median age was 3 years (range, 2–7 years), and median body weight was 13.1 kg (range, 8.5–31 kg).

### Hematologic Testing

Hematocrit and platelet count results are summarized in Table 1. One dog in the prednisone group had a mild thrombocytopenia (143,000 platelets/μL) at baseline, but platelet clumping was present. The following day, the platelet count was within the reference interval based on both automated count and manual review of a blood smear. Platelet counts and hematocrits otherwise were unremarkable for all time points. Platelet count did not differ significantly by treatment group, sampling time, or treatment-by-time. Hematocrit differed significant by sampling time [*F*(numerator degrees of freedom, denominator degrees of freedom) _(2,40)_ = 15.1, *P* < 0.001] but not treatment group or treatment-by-time. *Post-hoc* analysis revealed sampling time differences were due to significantly lower values on day 28 compared with baseline and day 14 (*P* < 0.001 for each).

**Table 1:** Mean ± standard deviation platelet counts and hematocrits for 24 healthy dogs administered placebo, aspirin with placebo, prednisone with placebo, or combination prednisone and aspirin therapy for 28 days.

	**Baseline**	**Day 14**	**Day 28**
**Platelet Count ( × ** **10**^**3**^**/μL)**			
Placebo	291 ± 78^a^	275 ± 53^a^	244 ± 53^a^
Aspirin	284 ± 73^a^	298 ± 113^a^	300 ± 89^a^
Prednisone	285 ± 60^a^	280 ± 48^a^	281 ± 74^a^
Prednisone and Aspirin	230 ± 69^a^	259 ± 94^a^	271 ± 87^a^
**Hematocrit (Percent)**			
Placebo	54.7 ± 5.8^a^	56.7 ± 3.5^a^	53.5 ± 4.8^b^
Aspirin	50.2 ± 8.3^a^	50.3 ± 6.8^a^	45.5 ± 6.9^b^
Prednisone	53.8 ± 3.8^a^	55.5 ± 4.4^a^	52.8 ± 5.4^b^
Prednisone and Aspirin	53.3 ± 3.5^a^	50.5 ± 6.1^a^	48.2 ± 4.3^b^

*Reference intervals: Platelet count = 170–400 × 10^3^/μL, hematocrit = 36–60%*.

*Results that do not share a superscript letter differed significantly (P <0.001) on post-hoc analysis*.

**Figure 1:**
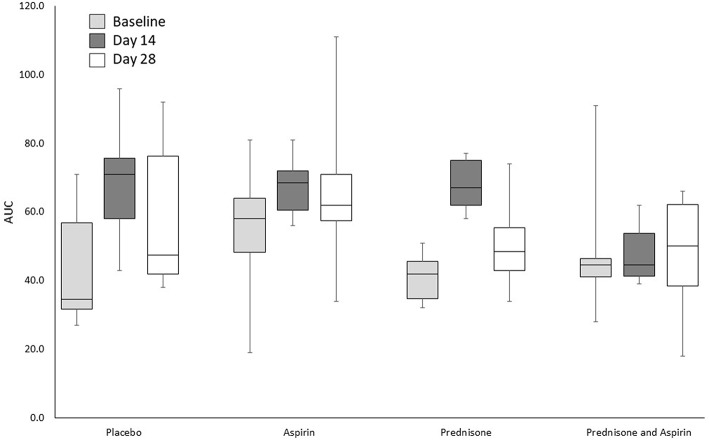
Impedance aggregometry AUCs for 24 healthy dogs administered placebo, aspirin with placebo, prednisone with placebo, or combination prednisone and aspirin therapy for 28 days. There were no significant differences in treatment group or treatment group-by-time interaction, but there was a significantly higher AUC on day 14 compared to baseline (*P* < 0.001) and day 28 (*P* = 0.048). The box and whiskers plot demonstrate the median (line), interquartile range (box), and total range (whiskers).

**Figure 2:**
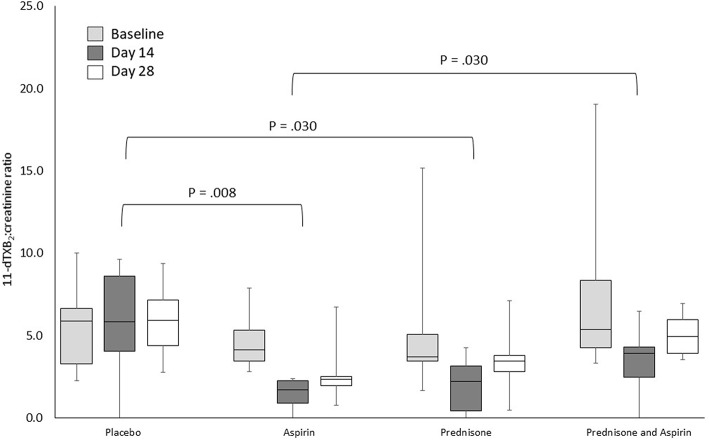
Urine 11-dTXB_2_:creatinine ratios for 24 healthy dogs administered placebo, aspirin with placebo, prednisone with placebo, or combination prednisone and aspirin therapy for 28 days. Ratios differed significantly by treatment group (*P* = 0.024) and sampling time (*P* < 0.001), but not treatment-by-time. The brackets indicate the significant differences between treatment groups. The box and whiskers plot demonstrate the median (line), interquartile range (box), and total range (whiskers).

The AUC for whole-blood aggregometry are presented in Figures 1, 2. Results differed significantly by sampling time [*F*_(2,40)_ = 10.2, *P* < 0.001] but not treatment group or treatment-by-time. This was due to significantly higher AUC on day 14 compared to baseline (*P* < 0.001) and day 28 (*P* = 0.048), and the AUC was significantly higher on day 28 when compared to baseline (*P* = 0.018) (Figure 1). Deviation in the mean AUC did not differ significantly by treatment group, sampling time, or treatment-by-time.

On day 14, there was 1 dog in the aspirin group and 1 dog in the placebo group classified as an aspirin responder. On day 28, there were two dogs in the aspirin group and one dog in the prednisone/aspirin group that were classified as an aspirin responder. Fisher's exact test revealed no significant relationships between aspirin responder status and treatment on day 14 or 28.

### Urine 11-dTXB_2_-to-creatinine Ratio

The urine 11-dTXB_2_-to-creatinine ratios for all treatment groups at all sample time points are presented in Figures 1, 2. Rank transformation was required prior to statistical analysis of urine 11-dTXB_2_-to-creatinine ratios. Urine 11-dTXB_2_-to-creatinine ratios differed significantly by treatment group [*F*_(3,20)_ = 3.9, *P* = *0.0*24] and sampling time [*F*_(2,40)_ = 8.7, *P* < 0.001], but not treatment-by-time. *Post-hoc* analysis revealed urine 11-dTXB_2_-to-creatinine ratios were significantly higher when comparing the placebo group with the aspirin group (*P* = 0.008), the placebo group with the prednisone group (*P* = 0.030), and the prednisone/aspirin group with the aspirin group (*P* = 0.030). Urine 11-dTXB_2_-to-creatinine ratios did not differ significantly between the placebo and prednisone/aspirin groups, prednisone and aspirin groups, and the prednisone and prednisone/aspirin groups (Figure 2). Finally, urine 11-dTXB_2_-to-creatinine ratios were significantly lower on day 14 than at baseline (*P* < 0.001) or on day 28 (*P* = 0.015).

## Discussion

Aspirin is commonly administered to prevent thrombus formation, but it is unknown if anti-platelet doses of aspirin counteract glucocorticoid-induced hypercoagulability. The results of our study suggests that sustained administration of an anti-platelet dose of aspirin does not significantly inhibit whole blood platelet aggregation when administered to healthy dogs alone or in combination with prednisone.

The mechanisms of exogenous glucocorticoid-induced hypercoagulability in dogs are unknown, but they might include decreased fibrinolysis, increased fibrinogen concentration, and decreased antithrombin activity (7, 8). In humans, chronic administration of exogenous glucocorticoids can cause a decrease in fibrinolysis and increase in fibrinogen concentration due to high plasminogen activator-inhibitor 1 activity (7, 28–30), but this has not been documented in dogs. Most studies evaluating the coagulation status of dogs during exogenous glucocorticoid administration have focused on measures of secondary or global hemostasis, such as prothrombin time, activated partial thromboplastin time, activated clotting time, fibrinogen concentration, antithrombin activity, thrombin-antithrombin complex, thromboelastography, and thrombin generation (7–9). Studies that have specifically evaluated primary hemostasis, or platelet aggregation, and thromboxane synthesis, have not been performed.

In previous studies, there have been contrasting results regarding platelet reactivity during the administration of immunosuppressive doses of prednisone. Similar to our study, Thomason et al. did not detect a significant difference in platelet function following administration of immunosuppressive doses of prednisone to healthy dogs (31). In contrast, despite using the same agonist to initiate platelet aggregation, Romao et al. demonstrated increased platelet aggregation in dogs treated with an immunosuppressive, but not an anti-inflammatory, dose of prednisone (32). Therefore, additional studies are required to better understand how glucocorticoids affect platelet function, especially when administered with anti-platelet therapy.

The results of our study did not demonstrate a significant decrease in platelet aggregation when dogs were treated with aspirin, either as a single agent or when combined with prednisone. These results were surprising because the aspirin dosage used in our study previously has been shown to consistently inhibit platelet function in dogs via two different platelet function analyzers (optical platelet aggregometry and the PFA-100) (14). Additionally, previous studies using lower dosages of aspirin (0.5 and 1 mg/kg q24h), have demonstrated platelet inhibition, although this inhibition was inconsistent (13, 17, 18). Based on previously established criteria (19), only 1 dog in the aspirin group and 0 dogs in the prednisone/aspirin group could be classified as an aspirin responder following 14 days of therapy. Following 28 days of drug administration, only two dogs in the aspirin group and one dog in the prednisone/aspirin group were classified as aspirin responders. One dog in the placebo group, on day 14, was classified as an aspirin responder without receiving any aspirin. The criteria used in our study to define aspirin response was based on a percent decrease from pre-treatment values. One possible explanation for this result is the variation with the platelet aggregometer. When using platelet aggregometry, small fluctuations in platelet function can occur, and these fluctuations could have contributed to classifying this dog as an aspirin responder. This is the first study to evaluate this combination of medications for an extended period of time, which could have contributed to the differences in results compared to previous studies (14, 18, 19).

Another potential explanation for these results is the use of the multiple-electrode impedance aggregometer. Minimal change was noted in platelet function of healthy dogs treated with an aspirin dosage of 1 mg/kg q24h for 7 days when platelet function was analyzed using the same multiple-electrode aggregometer using collagen as an agonist (19). Additionally, using the same parameters to define aspirin response, Haines et al. only identified 1 dog as an aspirin responder on day 3 of drug administration and 0 dogs on day 7. When samples were analyzed using conventional optical and impedance aggregometry, however, 13 of 16 dogs were classified as aspirin responders on both days 3 and 7 of therapy. One difference between Haines et al. and our study was the agonist used to initiate platelet aggregation, as we used arachidonic acid. A recent study showed that when using this multiple-electrode aggregometer, arachidonic acid provided the most consistent results when blood was treated with acetylsalicylic acid (24). Regardless of agonist, the multiple-electrode aggregometer might not be the ideal methodology for the assessment of aspirin-induced platelet dysfunction. Additional studies using optical aggregometry are indicated to better elucidate the impacts of sustained and/or concurrent prednisone and aspirin administration on platelet function.

One other possible explanation for our results was the use of a compounded aspirin product. This was done to provide accurate dosing, in keeping with prior studies and clinical practice (1, 15, 18, 19). To maximize efficacy of the compounded drug, all USP standards for compounding, including the use of a commercial aspirin product as the source material, were followed. Because plasma acetylsalicylic acid concentrations were not determined, altered bioavailability and/or product deterioration cannot be ruled out as a potential contributor to the results. However, we consider this unlikely given the significant association between aspirin administration and gastrointestinal bleeding in the related study, as well as the presence of new ulcers at day 28, in dogs administered aspirin (20).

Although treatment group and treatment-by-time interactions were not significant for AUC, AUC increased significantly over time (Figures 1, 2). This could reflect biological variability, although enhancement of platelet aggregation secondary to prednisone administration cannot be ruled out as a contributor to changes in two of the groups. It is also possible that recurrent anesthesia and/or endoscopy performed to evaluate gastrointestinal bleeding for the related study (20) caused platelet hyperactivity, confounding the results of this study. In our study, blood samples for platelet analysis were collected before the administration of any anesthetic medication and performance of endoscopy, and there was at least a 14-day recovery period before the collection of the next blood sample. Additionally, the anesthetic protocol used in the related study (acepromazine, butorphanol, and isoflurane) has been shown to have no significant effect on platelet function (33–35). Alternatively, these changes in the AUC on days 14 and 28 could reflect variability in the aggregometer or aggregometric data.

Aspirin irreversibly inhibits platelet function by inhibiting the cyclooxygenase (COX) enzyme and preventing the conversion of arachidonic acid to biologically active eicosanoids, prostaglandins, and TXA_2_, which are necessary for normal hemostasis (36). TXA_2_ primarily is produced by platelets and triggers platelet activation and vasoconstriction (37). Once synthesized, TXA_2_ is rapidly converted to stable metabolites, such as 11-dTXB_2_ and 2,3-dinor-thromboxane B_2_, that are eliminated in the urine and considered to be reliable markers of systemic TXA_2_ production (25, 26, 38). About one third of dogs have platelets that are sensitive to the activating effects of thromboxane (39, 40). Platelet activation through additional pathways and receptors could provide an additional explanation for why there was minimal inhibition of platelet function in our study.

Thromboxane, even if it does not affect all canine platelets, could still stimulate vasoconstriction. In our study, there was a 68% reduction in thromboxane synthesis on day 14 in the aspirin group, more than double the reduction found in a prior study (30%) despite use of the same aspirin dosage (14). As previously discussed, one potential explanation for the differences between studies is differing durations of drug administration. McLewee et al. administered aspirin for 7 days, while our study administered aspirin for 28 days, suggesting a potential progressive decrease in thromboxane synthesis. Dudley et al. previously demonstrated a progressive decline in thromboxane synthesis over 10 days of aspirin administration (18). Interestingly, in the aspirin group of our study, thromboxane synthesis appears to increase between days 14 and 28 such that day 28 values were reduced only 40% compared to baseline. One potential explanation for an increase in thromboxane concentration during chronic aspirin administration is compensatory upregulation of platelet COX-1 and/or COX-2 expression in response to a decrease in thromboxane concentration (18).

Prednisone, similar to aspirin, has anti-inflammatory properties that would inhibit the conversion of arachidonic acid to prostaglandin synthesis, including TXA_2_. Our study did not detect a difference in thromboxane synthesis between the aspirin and prednisone groups, suggesting that both of these medications had an inhibitory effect on thromboxane synthesis. As expected, thromboxane concentrations in both the aspirin and prednisone groups were significantly decreased compared to the placebo group. The results of our study contrast with results of a prior study in healthy dogs administered the same prednisone dose and using the same assay to measure 11-dTXB_2_ (31). In that study, an 80% increase in thromboxane concentration was noted after administration of the same prednisone dosage for 7 days to healthy dogs, although the increase was not consistent among dogs. It is possible that prednisone causes an initial increase in thromboxane concentration that wanes during chronic administration.

Although prednisone- and aspirin-induced inhibition of thromboxane synthesis was anticipated to be cumulative, thromboxane synthesis was significantly higher in the prednisone/aspirin group compared to the aspirin group in this study. It is possible that co-administration of the medications erodes inhibitory effects due to overlapping mechanisms of action. Although greater when prednisone and aspirin were used concurrently, thromboxane concentrations for both groups still were below pre-treatment concentrations, suggesting that combination therapy retains some anti-thrombotic benefits, such as vasodilation.

The aspirin dose used in this study was greater than that used in previous studies (7, 8, 18, 19). This dose was selected because of the potential for consistent inhibition of platelet function (14). However, the risk of gastric ulceration and hemorrhage increases with increases in the aspirin dose (16, 17). Glucocorticoids also have been shown to cause gastrointestinal hemorrhage and ulceration in healthy dogs (41–44). Co-administration of 2 ulcerogenic drugs, aspirin and prednisone, could increase the risk of gastrointestinal hemorrhage. Although not accompanied by changes in clinical signs (attitude, food intake, vomiting, fecal score), dogs administered prednisone, aspirin, and prednisone/aspirin in this study had significantly higher gastric endoscopic mucosal lesion scores due to increased punctate erosions, invasive erosions, and ulcers (20). Concerningly, scores were significantly higher on day 14 for dogs administered combination therapy, although severe lesions also were identified on day 28 and in dogs administered aspirin or prednisone therapy alone.

In whole blood aggregometry, dramatic decreases in the hematocrit can have a significant impact on the assessment of platelet function. A statistically significant decrease in hematocrit was noted on day 28 of this study, although hematocrit remained within the reference interval for all dogs. Gastric lesions could have contributed to this mild decrease in hematocrit, or it could have reflected biological variation. Regardless of cause, it is unlikely that the change in hematocrit noted in this study was large enough to meaningfully affect the aggregometric results.

There were several additional limitations to our study. Our study utilized healthy dogs with no evidence of hypercoagulability. Dogs with naturally-occurring disorders, hypercoagulability, and/or hyperactive platelets might respond differently to aspirin and prednisone. The sample size calculation indicated 6 dogs per group should be adequate to detect main effects, but that calculation was based on data collected using a different methodology and which differed substantially from the results ultimately obtained in this study. Enrollment of a larger sample size could have yielded different results. Additionally, only one instrument was used to assess platelet function; including multiple measurements of platelet function could have improved the assessment of platelet function. The instrument used in our study was an impedance aggregometer, which analyzes platelet function in fresh whole blood. Optical aggregometry, which uses platelet rich plasma, and flow cytometry might have provided different insights into platelet function. Furthermore, our study only evaluated two time points during drug administration, limiting comparison to prior work.

Another limitation to our study was that three techniques were used to collect urine for urine 11-dTXB_2_ quantitation, and it is possible that the lack of consistency in the collection technique could have influenced the 11-dTXB_2_-to-creatinine ratios. Additionally, blood and urine were collected within 4 h of drug administration, but sample collection was not performed at the exact same time point for each dog. Because aspirin irreversibly inhibits platelet function and therapeutic monitoring was first performed after 14 days of administration, the platelet function should be inhibited, this small difference in timing would be anticipated to have minimal effect on platelet function testing results. Additionally, previous studies in dogs have shown that the change in platelet function when treated with anti-platelet doses of aspirin are gradual, lacking dramatic changes during a short period of time (18, 19).

An additional limitation to our study was the assessment of only 1 thromboprophylactic agent. Anticoagulants, such as heparin and rivaroxaban, are considered the preferred anti-coagulant therapy in dogs with IMHA (5, 12). Unfortunately, these medications are expensive and/or require injectable administration multiple times per day, which is not feasible for many clients given current guidelines recommend continuation of thromboprophylaxis 6 weeks beyond discontinuation of glucocorticoid therapy in dogs with IMHA (5). Thus, oral anti-platelet therapy remains 1 of the most affordable, long-term thromboprophylactic therapies and can be an effective thromboprophylactic therapy in dogs with IMHA (5, 12). Additional studies would be required to assess the effectiveness of anticoagulant and non-aspirin anti-platelet therapies to counteract glucocorticoid-induced hypercoagulability.

Dogs with IMHA are predisposed to developing thromboembolism, and the administration of glucocorticoids might contribute to a hypercoagulable state. Our study indicates that sustained administration of aspirin at a dosage of 2 mg/kg, PO, q24h and/or immunosuppressive glucocorticoids does not significantly inhibit platelet aggregation. Additional studies using a wider array of platelet function testing methodologies in hypercoagulable dogs need to be performed.

## Data Availability Statement

The datasets generated for this study are available on request to the corresponding author.

## Ethics Statement

The animal study was reviewed and approved by the University of Tennessee, College of Veterinary Medicine Institutional Animal Care and Use Committee (protocol number 2283) and was in compliance with the requirements of a facility accredited by the American Association for Accreditation of Laboratory Animal Care.

## Author Contributions

JT, JP, and JW developed the hypothesis and study design, interpreted, and analyzed the results. AM and JW organized and conducted the experiment. JT, AM, JP, and JW wrote and revised the manuscript.

### Conflict of Interest

The authors declare that the research was conducted in the absence of any commercial or financial relationships that could be construed as a potential conflict of interest.

## Abbreviations

11-dTXB_2_: 11-dehydro-thromboxane B_2_
AUC: area under the curve
COX: cyclooxygenase
IMHA: immune-mediated hemolytic anemia
TXA2: thromboxane A_2_.
